# Assessing fall risk in osteoporosis patients: a comparative study of age-matched fallers and nonfallers

**DOI:** 10.3389/fdgth.2024.1387193

**Published:** 2024-07-10

**Authors:** Seong Hyun Moon, Krupa B. Doshi, Thurmon Lockhart

**Affiliations:** ^1^ Locomotion Research Laboratory, School of Biological and Health Systems Engineering, Arizona State University, Tempe, AZ, United States; ^2^ Division of Endocrinology, Mayo Clinic, Scottsdale, AZ, United States

**Keywords:** osteoporosis, falls, elderly, postural stability, physical activity level, age/gender-matched

## Abstract

This study aimed to investigate sway parameters and physical activity level of the age/gender-matched older adults with osteoporosis faller and nonfaller patients. By examining these factors, our objective was to understand how these faller and nonfaller groups with osteoporosis differed particularly in terms of balance capabilities and their impact on physical activity levels. We recruited 24 patients with osteoporosis: 12 who reported a fall within a year before recruitment (fallers) and 12 without falls (nonfallers). Given the close association between biochemical markers of musculoskeletal health such as serum calcium, parathyroid hormone (PTH), Vitamin D, and renal function, we compared these markers in both groups. As a result, elderly individuals with osteoporosis and with a history of falls within the preceding year indicated significantly higher sway velocity (*P* = 0.012*), sway area (*P* < 0.001*), and sway path length (*P* = 0.012*). Furthermore, fallers had significantly lower calcium (*P* = 0.02*) and Parathyroid hormone (PTH) (*P* = 0.02*), as well as higher Alkaline Phosphatase (ALP) (*P* = 0.02*) as compared to nonfallers despite similar vitamin D and creatinine levels. In conclusion, diminished biochemical factors in the osteoporosis faller group could possibly cause postural instability resulting in lower physical activity levels in the osteoporosis fall group and increasing the risk of falls.

## 1 Introduction

Falls and fall-related injuries pose a substantial medical and economic burden for elderly adults, emerging as a growing public health concern. Globally, fall related accidents are responsible for over 680,000 deaths and approximately 37 million annual healthcare visits (1)*.* In 2018, among adults aged 65 years and older, unintentional falls and related injuries were responsible for approximately 90% of 2.4 million emergency room visits (2)*.* Fall-related fractures drastically lessen an individual's quality of life, as they may not be able to get around for months or years after the fracture. Falls are also the most common reason for older persons being forced to transition from independent living to assisted care (3). This intricate relationship between falls and their result provides insight into the increased fracture risk associated with osteoporosis in older adults. Osteoporosis is a pervasive skeletal disorder that lowers bone mineral density and increases bone fragility, ultimately leading to an exponential increase in fracture risk in elderly adults (4). The most common fractures associated with osteoporosis occur in the hip, spine, and wrist. 95% of the distal forearm, 75% of the proximal humerus, and 25% of vertebral fractures are the result of osteoporosis-related falls (5, 6). Amongst these, hip fractures are the most serious, and have the most debilitating outcome of osteoporosis-related fractures in the elderly. In fact, after sustaining a hip fracture, a significant majority of individuals experience an acute decline in their mobility, ability for self-care, and participation in Activities of Daily Living (ADL) (7). Thus, hip fractures are a common reason for elderly adults to transition from independent living to dependent living with long term assisted care, despite successful surgical repair (8, 9). Additionally, among various fractures associated with osteoporosis, hip fractures have the strongest association with increased mortality. Studies indicated that approximately 20% of individuals who experienced a hip fracture die within the first year, and this risk continues to compound for the next several years (10). Furthermore, as individuals age, the fall frequency rises due to the coexistence of numerous chronic medical conditions that affect gait, posture stability, and physical activity levels. These conditions include the presence of a peripheral vestibular disorder, visual impairment, development of medical conditions that affect the neuromuscular system such as peripheral neuropathy or Parkinson's disease, muscle loss, and conditions that lead to changes in curvature of the spine or the weight-bearing axis (11–18). For example, age-related impairments of sensory systems, such as the vestibular and visual systems, cause loss of balance and spatial orientation, and reduction in acuity, depth perception, and peripheral vision, thus increasing the vulnerability of older adults to falls (19). Fall risk in women starts as early as in the mid-40s, coinciding with perimenopausal transition (20) and fall risk continues to increase throughout the lifetime for both genders, where men with lower testosterone levels are depicted as prone to fall risk (21), and more than 50% of women over the age of 85 suffer for at least one fall accident (22). Thoracic kyphosis, a consequence of osteoporosis spine fracture, is associated with lower muscle strength of trunk extensors, as well as impairment of joint position sense, and has contributed to an increased risk of future falls (16). Various issues could cause older osteoporosis patients to fall, such as poor gait stability (23, 24) and physical activity levels derived from the ADL (17, 25, 26). Physical activity level is an important factor that influences fall risk, with past studies indicating that individuals who exercise more have a lower fall rate (27). Similarly, individuals who performed low physical activity levels demonstrated significantly higher rates of severe falls compared to those who had moderate/high activity levels (18). Therefore, evaluating the physical activity level of osteoporosis patients, those who have fallen or not is an important factor that represents a significant indicator of the fall risk and has the potential of prevention.

More importantly, the essential factor that leads osteoporosis patients to fall is closely linked to postural stability control issues, where postural stability indicates the capability of an individual to control their center of pressure (COP) to balance themselves to avoid falls. Prior studies have depicted that postural instability is caused by osteoporosis, which originates from lower extremity muscle atrophy that leads to fall accidents (16). Generally, postural sway was utilized to assess the objective capability of maintaining the subject's posturography. Previous research has presented that elderly osteoporosis patients have a lower capability of controlling postural stability, which demonstrates the trend of low COP velocity, and higher sway area profile (8, 28). Therefore, investigating the postural sway and physical activity levels of osteoporosis faller and nonfaller groups is necessary to indicate who is more prone to fall and mitigate the risk of bone fracture. Moreover, age/gender-matched comparison of faller and non-faller in osteoporosis patients is essential because it minimizes age-related confounds, such as muscle, bone density, vestibular, and vision deterioration, where these aspects significantly differ among the various ages and dictates the capability of postural stability (14). The objective of this study is to analyze the disparities between fallers and non-fallers age/gender-matched osteoporosis patient's postural stability, and physical activity levels, and to elucidate how the fall status leads to a decrease in both the postural stability and physical activity levels.

## 2 Material and methods

### 2.1 Participants

To be included in the fall group, participants had to have fallen once in the year before they entered the study. To be included in the nonfall group, participants could have no falls within the year previous to study entry. A total of 24 elderly individuals with a diagnosis of osteoporosis (12 fallers, 12 nonfallers) who were living and ambulating independently participated in the study (Table 1). The groups were age and gender-matched such that there were 2 male and 10 female participants in each group. We excluded patients with a history of fractures not due to osteoporosis (such as pathologic fractures due to cancer metastases) and major comorbid conditions (such as dementia or visual problems). A research affiliate followed the participant recruitment protocol and asked eligible patients whether they were interested in being part of the study. If the patient agreed to participate, a physician discussed the study with the patient and answered all relevant questions. Participants were enrolled after written informed consent was obtained. The research was approved by the Mayo Clinic IRB and Arizona State University IRB. All research was performed in accordance with relevant guidelines and regulations.

**Table 1:** The subject's anthropometry data of the faller and nonfaller older adults with osteoporosis.

	Mean (SD)		*P*-Value
	Faller	Nonfaller	
Sample Size (Male/Female)	12 (2/10)	12 (2/10)	
Age, year	74.7 (8.7)	73.7 (7.7)	0.922
Height, cm	162.1 (6.1)	163.7 (7.6)	0.57
Weight, kg	66.1 (15.0)	65.3 (13.7)	0.89
BMI, kg/m²	25.5 (6.3)	24.4 (5.1)	0.64

The mean and standard deviation of faller and nonfaller age were 74.7 (8.7)/73.7 (7.7) (*P* = 0.922), height, was 162.1 (8.6) cm/163.7 (7.6) cm (*P* = 0.57), the weight was 66.1 (15) kg/65.3 (13.7) kg *P* = (0.89), Body Mass Index (BMI) 25.5 (6.3) kg/m²/24.4 (5.1) kg/m *P* = (0.64).

### 2.2 Study design

**Figure 1:**
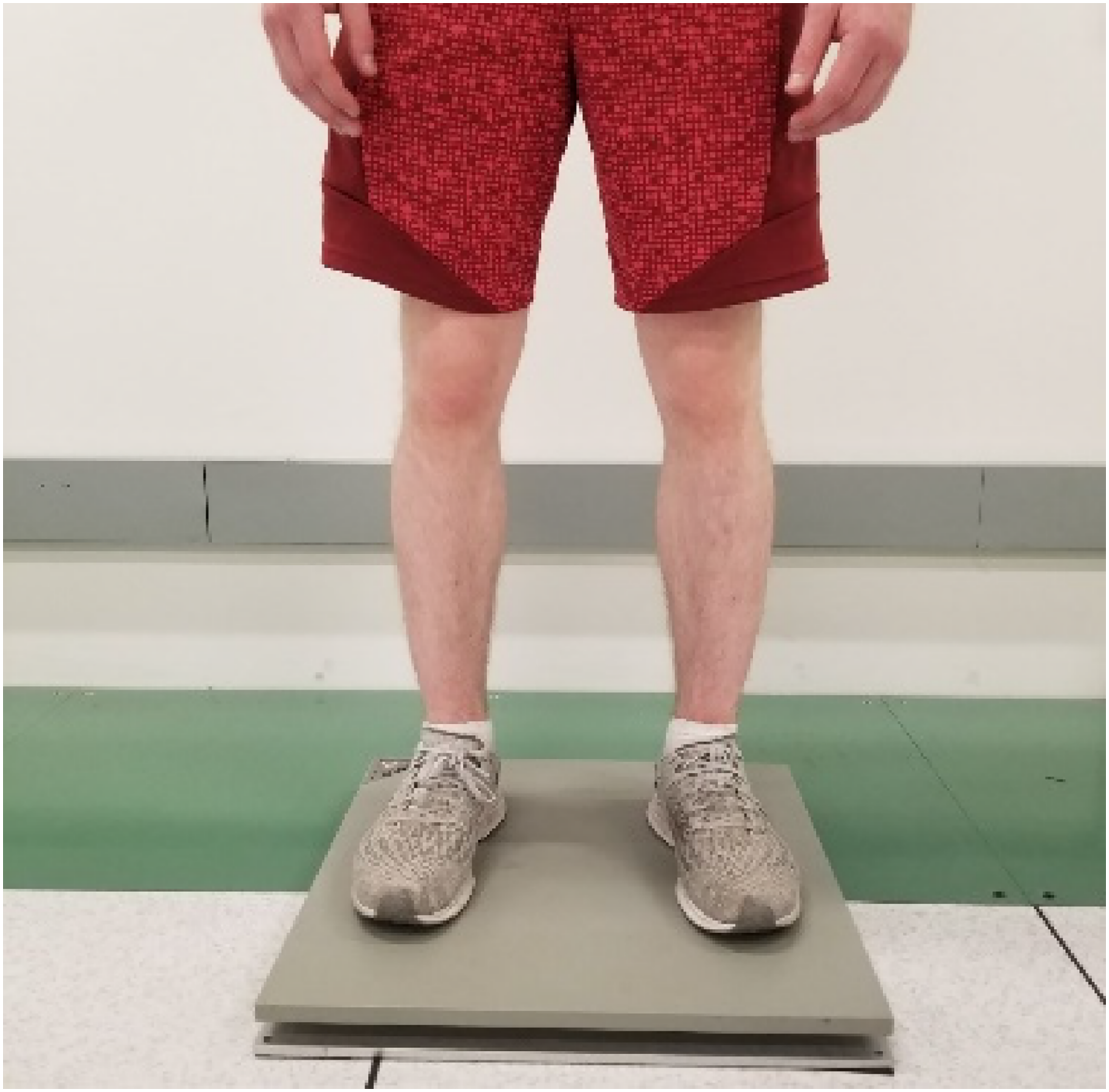
The subject standing on the AMTI portable force plate performing the postural stability testing.

Postural stability was assessed in a clinical environment and after the patient completed their outpatient clinic visit at Mayo Clinic, Arizona, Again Division of Endocrinology (13400 E Shea Blvd, Scottsdale, AZ, 85259), The environment was controlled for adequate light emission and ambient temperature. To assess postural stability, fallers and nonfallers were asked to maintain their normal balance with feet kept shoulder-width apart, and hands comfortably resting on their sides (Figure 1). The duration of this stability testing was measured for 60 s. During this time individuals were asked to concentrate their attention on the tape on the wall for visual assistance. In addition, they were instructed to avoid verbal communication to reduce the risk of head/trunk movement, which may influence postural sway measurements. Postural stability was assessed using AccuGait-O portable force plates (AMTI, Watertown, Massachusetts, USA; dimension: 502 mm × 502 mm × 45.5 mm; weight: 11.4 kg; sampling frequency:1,000 Hz). We included sway velocity, area, and path length as dependent variables for postural stability. The independent variable was the experience of previous ground-level falls as reported by the patient to the treating physician within a year from the date of the consent form.

### 2.3 Data analysis

#### 2.3.1 Postural stability collection and analyses

To measure postural stability utilizing the force plate data, fundamental assessment COP sway area, velocity, and total path length were computed. The computational analyses were conducted in MATLAB (MATLAB 2020b, The MathWorks, Inc., Natick, Massachusetts, United States). To analyze the sway area Equation 1 from the postural stability, the mean sway radius was calculated with anterior/posterior and medial/lateral movement of the center of pressure divided by the sample of data points (n) and multiplying the result by pi (π). Additionally, the COP sway path length Equation 2 was computed with the summation of Euclidean distance among the data points assessed throughout the total sway period. Sway velocity Equation 3 was calculated with sway path length divided by the total sway period.

(1) $$\mathrm{Sway}\;\mathrm{Area}\;(cm^{2})={\left(\frac{\sqrt{x^{2}+y^{2}}}{n}\right)}^{2}*\pi$$

(2) $$\mathrm{Sway}\;\mathrm{Path}\;\mathrm{Length}\;(\mathrm{cm})=\sum_{n-1}^{n}\sqrt{{\left(x_{n}-x_{n-1}\right)}^{2}\;+\;{\left(y_{n}-y_{n-1}\right)}^{2}\;}$$

(3) $$\mathrm{Sway}\;\mathrm{Velocity}\;(\mathrm{cm}/s)=\frac{1}{t}\;*\;\mathrm{Sway}\;\mathrm{Path}\;\mathrm{Length}$$

Consequently, by utilizing this methodology (29, 30), it is possible to compute postural stability, where it indicates the capability of subject maintaining their center of mass within the defined boundaries, such as stability limit (31). The assessment includes parameters such as sway area, path, and velocity. These elements are critical factors in determining the fall risk of elderly individuals with osteoporosis.

#### 2.3.2 Physical activity data assessment and analyses

Our study conducted 72 h of longitudinal data assessment at the participant's residences. Each participant was required to wear the IMU device on their sacrum area for consecutive 3 days and simultaneously document their Activities of Daily Living (ADL). For the ADL journal, patients were required to log their daily activities, and classified into four primary movements, which were sitting, standing, walking, and laying down. In addition, the inquiry involving the detailed location where the activities were executed was recorded. Patients were directed to record these activities in one-minute intervals, and researchers utilized the journal to examine the coordination between IMU data and the activities that were executed by the subject. The device that was used to collect the ADL data was Dynaport MM+ (Motion Monitor+, McRoberts BV, The Hague, Netherlands. This sensor's specification had a dimension of 85 × 58 × 11.5 mm, a weight of 55 grams, and a sampling frequency capability of 100 Hz. This transforms human activity data into the raw acceleration signal form, the X, Y, and Z coordinate acceleration was converted into resultant acceleration. This data was preprocessed with MATLAB, using high and low-pass Butterworth filter, eliminating the unnecessary noise signals that could have been recorded during the data assessment. Subsequently, a 1-Hz threshold cut-off frequency was implemented to indicate the dynamic and static physical activity level of each patient. This algorithm allowed us to compare the osteoporosis faller and nonfaller patient's ADL physical activity levels (17).

## 3 Results

The subject's anthropometric data was meticulously matched, confirming equal gender distribution into fall and nonfall groups, with the sample size of 2 males and 10 females in both groups. In addition, the mean age of fallers was 74.7 years, and the nonfaller group was 73.7 years, indicating a minimum of 1-year difference. The average height (cm) of both groups was also closely aligned, where the faller's height was 162.1 cm and the nonfaller's was 163.7 cm, with merely 1.6 cm distinction (*P* = 0.57). Similarly, the mean weight (kg) was 66.1 kg for the fallers and 65.3 kg for the nonfaller group, with a 0.8 kg variance (*P* = 0.89). Lastly, the BMI (kg/m²) of the faller was 25.5 and the nonfaller was 24.4, depicting the deviation as 1.1 (*P* = 0.64). These similar anthropometric characteristics allow for identical comparisons between the two groups. Our results showed numerous significant differences between both cohorts and are presented in Table 2. The postural stability results showed that the fallers had significantly higher sway velocity (1.82 cm/s) as compared to nonfallers (1.22 cm/s) (*P* = 0.012*). Fallers also have a significantly higher sway area (3.86 cm^2^) than nonfallers (1.74 cm^2^) (*P* < 0.001*). The sway path length was also higher in the fallers (90.84 cm) as compared to the nonfallers (60.93 cm), (*P* = 0.012*). Additionally, the dynamic level of the nonfaller (21.6%), (*P* = 0.004*) was significantly higher compared to the fallers. We observed several biochemical differences between the faller and nonfaller groups. Despite similar mean vitamin D (*P* = 0.57) and creatinine levels (*P* = 0.5), the mean serum calcium level was significantly lower in the faller group (9.34 mg/dl) compared to the nonfallers (9.7 mg/dl), (*P* = 0.02*). The mean parathyroid hormone level was higher in the faller group (71.94 pg/ml) than the nonfaller group (36.97 pg/ml) (*P* = 0.02*). Similarly, the mean total alkaline phosphatase was also higher in the faller group (84 IU/L) than the nonfaller group (61.7 IU/L), (*P* = 0.02*).

**Table 2:** The osteoporosis faller and nonfaller subjects’ measurement variables.

	Mean (SD)		*P*-Value
	Faller (*n* = 12)	Nonfaller (*n* = 12)	
Total calcium level (mg/dl)	9.34 (0.4)	9.7 (0.3)	0.02*
Vitamin D (ng/ml)	38.9 (14.0)	42.7 (16.4)	0.57
Creatinine (mg/dl)	0.93 (0.2)	0.86 (0.2)	0.5
Parathyroid hormone (pg/ml)	71.94 (39.3)	36.97 (12.0)	0.02*
Alkaline Phosphatase (IU/L)	84 (23.7)	61.7 (13.2)	0.02*
Sway Velocity (cm/s) [FP]	1.82 (1.01)	1.22 (0.48)	0.012*
Sway Area (cm^2^) [FP]	3.86 (2.08)	1.74 (0.53)	<0.001*
Sway Path Length (cm) [FP]	90.84 (50.70)	60.93 (24.13)	0.012*
Dynamic Physical Activity Level (%) [IMU]	8.66 (5.55)	21.6 (12.1)	0.004*

* Indicates *p* < 0.05.

## 4 Discussion

For older adults, postural stability determines how securely a person remains stable around their center of mass (COM) during the stance (32). Balance loss can occur during a quiet stance when the subject's COM moves outside of the range of their base of support (the area between their two feet). Lack of ability to maintain the balance is one of the major factors that increases fall risk (33). Also, the Center of Pressure (COP) is derived from the ground reaction forces, indicating the vertical projection of the COM and the rotational force applied to the ground from the feet (34). For a person to maintain balance during a quiet stance, they must be able to elicit appropriate postural strategies of balance. If they are unable to do so, the only action may be to progress to a more dynamic type of movement (a step or grab onto a nearby object) to avoid completing a fall. Multiple studies have attempted to identify differences in postural stability during dynamic movement between older adult fallers and non-fallers (35–39). In this study, we attempted to evaluate the difference in postural stability and physical activity level between faller and non-faller age/gender-matched osteoporosis patients and found significant differences with fallers demonstrating higher sway velocity, area, path length, and lower dynamic activity level as compared to nonfallers. By investigating these various postural stability parameters, such as, sway velocity designates the mean horizontal displacement covered by the COP movement in both the anterior-posterior and medial-lateral directions per total time series length of data.

The sway path length parameter presented a significant increase in the osteoporosis faller group. This factor signifies increased oscillations within the faller group and implies a reduced capability of maintaining balance compared to the non-faller group (39, 40). The Postural Sway path length represents the total distance covered from the subject during the quiet stance, where it quantifies the displacement of the Center of Pressure (CoP) in two dimensions (anterior-posterior and medial-lateral) based on the overall distance traveled (41). Where past studies have depicted that the faller group of COP path length was determined to have higher sway path length compared to the non-faller group (35, 42). Additionally, the sway areas have depicted a significantly higher in osteoporosis faller group. This observation depicts the range of sway area of the subject, demonstrating the limits of the subject's balance. This result determined that the limit of stability values deteriorated, and functional balance results were worse in the faller group compared to the non-faller group. The results from our study were supportive of the difference in the postural control amongst fallers and nonfaller osteoporosis subjects. Our results align with the outcome of Ucurum et al. where this research depicted that women with osteoporosis had higher anteroposterior oscillations, limiting their capability to maintain postural stability and maintain functional balance compared to the non-osteoporotic group (43). Additionally, the capability of controlling postural stability tends to degenerate with aging and disease (44, 45).

Osteoporosis patients tend to have less muscle strength that controls postural stability (11), which leads to a higher risk of getting a fracture due to low mineral density (5, 46). Furthermore, these patients have significantly higher fear of falling which indicates the lack of balance confidence, making them more prone to fall (47, 48). Correspondingly, aging balance disorder is closely related to the degradation of the vestibular apparatus, where its functionality is responsible for controlling the postural stability to maintain balance and allow a person to react immediately to unpredicted perturbations (49). Moreover, fall risk is proportional to the aging population, because older adults have limited functional postural control systems (44), where this instability develops from the deterioration of multi-sensory subsystems as people age (50). Older adults tend to develop focusing disorder, visual impairment (51), limited somatosensory capabilities (52), and degradation of vestibular function (53). Therefore, the results of our study indicated that older adults with osteoporosis in the faller group are prone to fall risk due to lower postural stability. This closely aligns with indicating importance the exceedingly high fall risk, which involves an increased possibility of falling due to loss of muscle mass, and a heightened potential for fracture risk. As a consequence, this fracture tends contribute significantly elevated the mortality rate (54).

Furthermore, various past studies have demonstrated that exercise can decrease the possibility of falling. Pereira et al. demonstrated a reduction in severe fall accidents resulting in significant injuries that decreased in individuals with moderate physical activity levels (76.2%) and high physical activity levels (57.5%) compared to the subjects with low physical activity levels (18). Another study has observed that subjects who perform higher exercise levels and activity level decreases the rate of falls by 23% (27). In addition, Skelton et al. have indicated that exercise and higher physical activity levels do improve postural stability (55), and characteristics of Fear of Falling (FOF) can be predominantly observed in frail older adults (56). Correspondingly, our result illustrated that the osteoporosis faller group demonstrates lower physical activity levels. This is due to the decreased maintaining the capability of postural stability, which has the potential of causing the reduction in the physical activity level. Thus, our result depicts that these two aspects are closely related, where less physical activity level shows a significant difference between osteoporosis faller and nonfaller, which could be primarily derived from postural instability. Additionally, biochemical indices such as calcium and Vitamin D status have been linked to a higher risk of falls in older adults with osteoporosis. In our study, the fallers had lower mean serum calcium and higher mean parathyroid hormone levels than nonfallers, while mean vitamin D levels and kidney function did not differ between the two. Factors that can lead to low serum calcium include a low dietary calcium intake or reduced intestinal calcium absorption. Low calcium intake and availability contribute to osteoporosis (57), and also predict significant muscle loss in adults (58) thus calcium deficiency increases the risk of osteoporosis, sarcopenia, and falls, serving as a catalyst for fractures. Low serum calcium, low Vitamin D levels, or elevated creatinine levels can lead to an increase in parathyroid hormone levels. However, in our study both groups had similar levels of vitamin D and creatinine, therefore we hypothesize that elevated parathyroid hormone level was likely due to low serum calcium.

In summary, osteoporosis is an intricate endocrinological disorder that influences balance capability (43), and result indicates that previously fallen osteoporosis patients have less postural stability and physical activity levels compared to non-fallers. Our study concludes that the fall group has depicted a significant decrease in postural stability, and lower activity level compared to the non-faller group. These stability and activity characteristics indicate that osteoporosis patients who have fallen previously are prone to recurrent falls, and this significantly increases issues for osteoporosis patients (who are already at a high risk of fracture due to low bone mass). Thus, early balance and physical activity assessment is required to identify and reduce the possible fall risk. Future studies should extend beyond the comprehensive analysis of the osteoporosis patient's postural stability and physical activity level and investigate the intervention effect of exercise training and physical therapy. Our analyses are based on one fall scenario wherein the fall incident is critical to osteoporosis patients who are recurrent fallers should also be observed for further understanding of postural control and activity of daily living. This multifaceted process will provide an in-depth evaluation of the osteoporosis patient's fall risk and contribute to significant awareness of fall prevention strategies and interventions.

## Data Availability

The datasets are not publicly available due to restrictions used under the license for the current study. There are available on reasonable request from the corresponding author.
